# Breast cancer surveillance in *BRCA* positive Sri Lankan women: health equity for a high-risk group at a limited resource setting

**DOI:** 10.1186/s12905-023-02797-z

**Published:** 2023-11-28

**Authors:** Udari Apsara Liyanage, Nirmala Dushyanthi Sirisena, Pushpika Chathuranga Deshapriya, Vajira Harshadeva Weerabaddana Dissanayake

**Affiliations:** Department of Anatomy, Genetics and Biomedical Informatics, Faculty of Medicine, University of Colombo, Colombo, Sri Lanka

**Keywords:** Cancer genetic screening, Hereditary breast cancer, Breast cancer risk, Screening mammography, South Asia

## Abstract

**Background:**

*BRCA1* and *BRCA2* pathogenic variants account for 90% of hereditary breast malignancies, incurring a lifetime breast cancer risk of 85% and 40–45% respectively, in affected individuals. Well-resourced health care settings offer genetic counselling and genetic screening for susceptible individuals, followed by intense breast cancer surveillance programmes for those identified at high risk of breast cancer. Such high standards of care are not available in countries with limited resources. This study assessed breast cancer surveillance behaviors among a cohort of *BRCA* positive Sri Lankan women.

**Methods:**

A retrospective case review of all patients diagnosed with pathogenic variants in *BRCA1* and *BRCA2 *genes from 2015 to 2022 at the Human Genetics Unit, Faculty of Medicine, University of Colombo was carried out followed by telephone interviews of the respondents. Patients who were not contactable, deceased, undergone bilateral mastectomy and males were excluded from the interview component of the study. Standard descriptive statistics were used to analyze the data using SPSS statistics version 25.

**Results:**

Only 25 patients were diagnosed during the study period:14/25 women responded (6/25 deceased, 3/25 non-contactable; 2/25 excluded). 71.4% (10/14) had performed breast self-examination during the preceding month; 35.7% (5/14) had a clinical breast examination (CBE), and 50% (7/14) had undergone a screening/diagnostic mammogram during the last one year. 28.5% (4/14) had undergone both mammography and CBE; 21.45% (3/14) mammogram only, 7.1% (1/14) had CBE only. 42.8%(6/14) had not undergone any surveillance(mammography, CBE or MRI). None had dual screening with mammogram and MRI. 85.71% (12/14) women expressed willingness to participate in a regular screening programme if made available.

**Conclusion:**

Fifty percent of *BRCA1/2* positive women in our study had not undergone annual imaging-based surveillance by mammography or MRI, and none had undergone annual dual screening with mammography and MRI, indicating inadequate breast cancer surveillance in this high-risk group.

## Background

Genetic predisposition accounts for 5–10% of all breast cancers [1, 2], primarily attributed to pathogenic variants in *BRCA1* and *BRCA2* genes, responsible for nearly 90% of hereditary breast cancers [1]. Women with pathogenic variants in *BRCA1* and *BRCA2* genes are considered a high-risk category for breast cancer having a lifetime risk of developing a breast malignancy of 85% and 40–45%, respectively [3]. High risk, as per the National Institute for Health and Care Excellence (NICE) UK guidelines, denotes a lifetime breast cancer risk exceeding 30% from age 20 onwards, while risk under 17% is considered average [4].

In well-resourced countries, genetic counseling and screening are offered to susceptible individuals identified through family or clinical history [1, 5] High-risk individuals receive preventive measures and intensified cancer surveillance measures starting at an earlier age than average risk population in USA, Europe, UK and in many other well-resourced settings including Singapore, Malysia and China [1, 5–7]. Dual screening with mammography and MRI is known to identify more breast cancers than either screening tool alone for this group of women [5]. American College of Radiologists (ACR) recommends annual digital mammographic screening staring from the age of 30 years, and annual breast MRI from 25–30 years of age, for women with genetic predisposition leading to increased risk of breast cancer [7]. For *BRCA 1/2* positive women, National Institute of Health (NICE), UK guidelines recommend offering annual surveillance MRI from 30–49 years and annual mammographic surveillance from the age of 40–69 years [5]. Continuing annual examination of the chest wall is recommended even after prophylactic mastectomy [1]. Some countries extend such intense surveillance to untested first-degree relatives and high-risk individuals based on family history [7, 8], highlighting the high standards of care offered to women at high risk of breast cancer in some parts of the world.

However, in limited resourced settings, particularly in low and lower-middle-income regions of Asia, opportunities for mammographic breast cancer screening are much less. The number of mammography units per one million women aged 50 to 69 years is reported as low as 0 in Bhutan, and 2.81 in Sri Lanka to 32.6 in Mongolia, compared to high-income (HI) countries such as, 127.6 in Singapore and 227.3 in Japan [9]. Many Asian low-income (LI) and lower middle-income (LMI) countries do not conduct organized national mammographic screening programs [9]. This is in consensus with the WHO position that population screening mammography for average risk women is not cost effective for limited resource settings with weak health systems (LI and LMI countries given as examples in the position paper) as opposed to well-resourced settings (e.g. most HI countries) [10]. Nevertheless, not offering organized surveillance for the high-risk individuals is questionable. The WHO position paper lacks clear guidelines for high-risk women [10], and globally, there is lack of locally adaptable guidelines for varying risk levels across socio-economic backgrounds [6].

Sri Lanka's age-standardized breast cancer rates have risen from 18.4 per 100,000 in 2005 to 33.5 in 2019 [11]. Free healthcare is offered in the state sector, and the national cancer control programme (NCCP) of Sri Lanka advocates early detection of breast cancer. Although the country lacks an organized national mammographic screening program similar to other resource poor countries in the region [12, 13], opportunistic breast screening services are available mostly focusing on breast awareness, self-breast examination (SBE), and clinical breast examination (CBE) [14]. Mammographic screening is recommended for the average risk category aged 50–69 years, at 2–3-year intervals, to be adapted only when adequate facilities exist [15].

Although low in number, state sector mammographic facilities are established across all provinces in Sri Lanka, primarily focusing on diagnostic services [16]. While a few dedicated breast clinics provide CBE and screening mammography on availability, by appointment via websites [14] or by onsite registration, no patient registration systems for screening invitations/reminders exist. The private sector, a key contributor to heath care provision in Sri Lanka, also offers opportunistic mammography. Most Sri Lankans pay out of pocket in the private sector, with limited health insurance coverage [17]. Breast MRI is available only in select hospitals.

High-risk women in Sri Lanka access opportunistic screening including mammographic facilities, similar to average-risk women. There are no dedicated national programs prioritizing their surveillance. No systematic high-risk women identification or registry exists. Although updated national guidelines acknowledge the need for intensified screening for *BRCA* variant carriers, giving options for annual multimodality surveillance referring to guidelines from HI countries, they lack guidance on resource-adaptive implementation [15]. Only a few centers offer genetic testing in Sri Lanka, with testing costs of 125,000 LKR (~ 390 USD) raising concerns about affordability.

In this context, our study assessed breast cancer surveillance behaviors among a cohort of Sri Lankan women genetically predisposed to breast cancer with *BRCA1* or *BRCA2* pathogenic variants. Our aim was to understand high-risk women's behaviors in the backdrop of limited-resource opportunistic screening in countries like Sri Lanka. This knowledge can guide better resource allocation, prioritize high-risk groups, and spark discussions on the necessity for more detailed guidelines and proactive referral systems.

## Methods

### Study setting and study design

A cross sectional observational study was carried out in a cohort of patients diagnosed with pathogenic variants in *BRCA1* and *BRCA2* genes from 2015 to 2022 period at the Human Genetics Unit (HGU), Faculty of Medicine, University of Colombo.

The HGU serves as the principal National Referral Center for offering genetic counselling and genetic testing in Sri Lanka, and receives referrals from the state and private sector from all over the country [18, 19] The center provides pre- and post-testing genetic counselling by clinical geneticists, free of charge to all registered patients, but genetic testing is carried out as a fee levying service. Post-test counselling involves a discussion on the genetic test results and their implications for the patient and family members, as well as medical management options, including cancer surveillance and treatment options. HGU maintains a de-identified cancer genomics database and confidential detailed clinic records of all patients registered in the center. At the initial visit at the HGU, each patient is given a registration number which can be used as the link to the clinical records of the patient. The de-identified database contains only the registration numbers of the patients along with their genomic data, no personal identifiable data are included in it. The personal identifiable data and contact details are contained only in the clinical records of patients which are kept under confidential cover. The patient registration number contained in the database was used in retrieving the clinical records of the patients.

### Study population, patient recruitment and ethical considerations

The study population included all patients diagnosed with pathogenic variants in *BRCA1* and *BRCA2* genes during the study period. Patients who had consented to undergo genetic testing and whose test reports were either *BRCA1* or *BRCA2* positive were selected from the de-identified database of the HGU using the patient registration numbers and their detailed clinic records were retrieved. Subsequently patients were contacted by telephone (see ethical considerations below). Patients who were not contactable, deceased, undergone bilateral mastectomy, and male patients were excluded from the relevant interview components of the study.

#### Ethical considerations

Ethical clearance was taken from the Ethics Review Committee, Faculty of Medicine, University of Colombo. Informed written consent for willingness to be contacted, and willingness to contribute one’s information from clinical records/databases for research purposes, is normally obtained from the patients during the pre-test counselling session prior to genetic testing. Further informed verbal consent was taken for this particular study when the patients were contacted over the phone. Patients were given the option to have more time to consider their willingness to participate in the study. Consenting patients were given the opportunity to decide on a preferable time to be contacted for data collection and a second phone call was made at their preferred time. Data was collected only by the authors (investigators). A simulated practice session was held prior to data collection to avoid/minimize any confusion or emotional trauma to the patient. Only the information that was not in the medical records, such as current income, screening behaviours, and details that had to be verified such as incomplete data fields were gathered at the telephone interview to reduce patient exhaustion. Family history was retrieved from the detailed pedigree documented in the clinic records to avoid emotional trauma in recalling family cancer history.

### Data collection process and data analysis

Data was collected from the HGU database, the clinic records and by telephone interview according to a pretested data collection form for each patient and entered into a database. Indication for referral, family history and medical information were collected from HGU records. Current socio demographic data, perceived risk of breast cancer, information on breast self-awareness and breast screening behaviors and willingness to attend a screening programme were inquired during the telephone interview.

Breast self-awareness was assessed by asking if participants observe their breasts in the mirror to check if they look normal, and by inquiring if they would consider seeking further assessment by a doctor or healthcare worker, to rule out breast cancer, if the following symptoms/signs were noted by the participant: nipple discharge (other than breast milk; especially a bloody discharge), change in size or shape of a breast, skin irritation such as redness, thickening or dimpling of the skin, swollen lymph nodes in the armpit, nipple problems such as pain or redness, swelling of a breast and breast pain occurring with the menstrual cycle. Practice of Self-Breast Examination was checked for the frequency, and inquiring who trained the patient on the technique. Screening behaviors with CBE, mammography and MRI were checked for frequency of attendance for individual modalities, and if participants had received surveillance by multiple screening tools such as dual screening by mammography and MRI.

Strong family history was defined as per recommendations made by 2019 updated NICE guidance, UK [20]. Standard descriptive statistics were used to describe the data using SPSS version 25 statistical software.

## Results

A total of 25 patients, belonging to 23 families were diagnosed as *BRCA1* or *BRCA2* positive from 2015 to 2022. Their ages and indications for genetic screening are shown in Table 1.

**Table 1:** Age and indication for genetic screening (*n* = 25)

**Age at genetic testing**	
Age range	27–72 yrs
Age in categories	
< 30 years	1(4%)
30–39 years	3(12%)
40–49 years	8(24%)
50–59 years	7(28%)
> 59 years	6(24%)
**Indications for referral to specialist genetic clinic**	
Family history and personal history of breast/ovarian cancer	8(32%)
Family history of breast /ovarian cancer with no personal history of cancer	2(8%)
Family history of multiple cancers other than breast /ovarian cancers	1(4%)
Personal history of multiple cancers (breast/ovarian), no family history	4 (16%)
Personal history of breast or ovarian cancer at < 45 years	2(8%)
Family screening for known *BRCA1/2* mutation carriers in family	3(12%)
Male breast cancer	1(4%)
Other	4(16%)

14/25 (56%) had family history of breast/ovarian cancer: 9/14 (64.2%) of this group had strong family history of breast or ovarian cancer, and 55.5% (5/9) individuals with strong family history had had a genetic diagnosis made after developing at least one personal breast/ovarian cancer, while one patient (1/9;11.1%) with strong family history received a genetic diagnosis after developing the second personal breast/ovarian cancer.

Only 14/25 (56%) participated in the telephone interviews, as 6/25 (24%) were deceased due to advanced breast/ovarian cancer, 3/25(12%) were not contactable and two (2/25;8%) were excluded as one patient had undergone bilateral therapeutic mastectomy and one was excluded due to male gender. Only one (1/6;16.6%) of the deceased patients had a strong family history of breast/ovarian cancer. One of the non-contactable patients had metastatic breast cancer at the time of genetic referral. All 14 participants were diagnosed as *BRCA1/2* positive at least one year before data collection.

Details of the patients who participated in the telephone interviews are shown in Table 2.

**Table 2:** Details of patients who participated in telephone interviews (*n* = 14)

Sociodemographic parameters	
Age	
Range	29–76 years
Age in categories	
< 30 years	1(7.1%)
30–39 years	2(14.3%)
40–49 years	6(42.9%)
50–59 years	1(7.1%)
> 59 years	4(28.6%)
Marital status	
Married	14(100%)
Educational level	
GCE O/L	6(42.9%)
GCE A/L	5(35.7%)
Higher education	3(21.4%)
Employed	
Yes	7(50%)
No	7(50%)
Family income	
Monthly > 100,000 LKR (approximately > 312USD)	7(50%)
Monthly 20,000–100,000 LKR (approximately 63-312USD)	7(50%)
Monthly < 20,000 LKR (approximately < 63USD)	0
Distance to the nearest mammographic facility from home	
< 25 km	14(100%)
> 25 km	0(0%)

### Perceived risk of developing breast cancer

The majority (11/14;78.6%) were aware that they are at increased risk of breast cancer compared to the rest of the population. However, none of the 14 respondents knew the actual lifetime risk of a *BRCA* positive woman developing breast cancer; 78% (11/14) said that they do not know, and the remaining 3/14 (21.4%) expressed figures between 1- 25%.

### Breast self-awareness and breast screening behaviors with CBE, Mammography and MRI

Breast self-awareness, SBE practices and breast screening practices and behaviours related to CBE, mammography and MRI are shown in Table 3.

**Table 3:** Practices and screening behaviours among participants (*n* = 14)

Practices and behaviors	Number	Percentage
***Breast self-awareness***		
Observe breasts in the mirror to check if they look normal	13	92.8
Symptoms/signs perceived as a reason to seek further assessment		
Nipple discharge other than breast milk (especially a bloody discharge)	13	92.8
Change in size or shape of a breast Skin irritation such as redness Thickening or dimpling of the skin Swollen lymph nodes in the armpit Nipple problems such as pain or redness Swelling of a breast Breast pain occurring with the menstrual cycle	13 13 13 13 13 13 12 6	92.8 92.8 92.8 92.8 92.8 92.8 92.5 46.1
BSE practices		
Performed BSE within the last month	10	71.4
Performed BSE, but more than one month back	2	14.3
Not performed BSE at all	2	14.3
***CBE for cancer surveillance***		
Underwent CBE within the last one year	5	35.7
Underwent CSE more than one year before	2	14.3
Not had CBE at all	7	50
***Mammography* within last one year**		
Underwent screening mammography	5	35.7
Underwent diagnostic mammography	2	14.2
Not undergone mammography (screening/diagnostic)	7	50
***Mammography* within last two years**		
Underwent screening mammography	7	50
Underwent diagnostic mammography	2	14.2
Not undergone mammography (screening/diagnostic)	5	35.7
***MRI after genetic diagnosis***		
Underwent screening MRI	1*	7.10
Underwent diagnostic MRI ***-***	0	0
Not undergone MRI (screening or diagnostic) at all	13	92.8

^*^had screening MRI 3 years back

Nine out of the twelve (75%) women who had performed BSE at some point of time in their lives were trained by healthcare staff and the others (3/12;25%) had self-learnt the procedure.

**Fig. 1:**
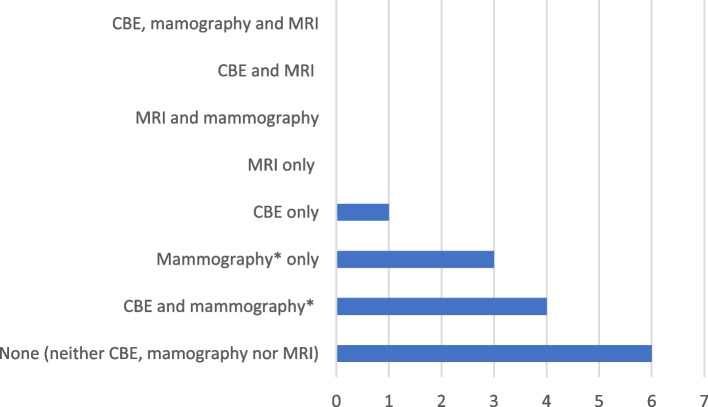
Combinations of surveillance tools used during the last one year (*n* = 14) *Both screening and diagnostic mammographic examinations are included (2/7 mammographic examinations were diagnostic – refer Table 3)

Annual Surveillance pattern relevant to the past one-year period is shown in Fig. 1.

Even when surveillance within the past two years was considered, none underwent screening MRI, neither as a single modality nor in combination with other modalities; 35.7% (5/14) had undergone dual surveillance with CBE and mammography, and 28.5% (4/14) had undergone mammogram only. 35.7% (5/14) had not undergone either CBE, mammogram, or MRI during the last two years.

### Mammographic surveillance based on age categories

**Fig. 2:**
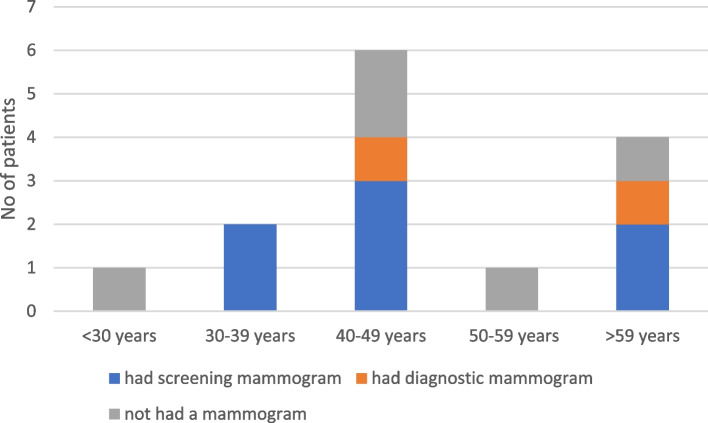
Mammography within last two years, in age categories (*n* = 14)

Out of the 7 women who had not had a mammogram within the last one year (refer Table 3), five were aged ≥ 40 years of age. The other two were aged 29 and 34 years. Age categories of the women who had screening mammography (*n* = 7;refer Table 3) within the last two years are indicated in colour code blue in Fig. 2.

Five of the above screening mammograms (5/7;71.4%) were done free of charge in the state sector while 2/7 (28.6%) were done in the private sector.

### Willingness to participate in a screening programme in future

85.71% (12/14) women expressed willingness to participate if a regular screening programme was made available. One woman said it is not required for her as she was already being followed up by private consultation and another woman did not respond to this question.

## Discussion

This study revealed that only 25 cases were diagnosed with germline pathogenic variants in the *BRCA1/2* genes during the study period at HGU. Considering that only a limited number of centers offer genetic testing services in Sri Lanka and given that over 3000 new cases of breast cancer are diagnosed annually in the country, with an increasing incidence rate [21], the number of only 25 cases identified at HGU can be considered a concerning low case number of the genetic diagnosis. Exploring the underlying reasons for the low case numbers may provide insights into the barriers to improving healthcare for high-risk individuals.

Obstacles such as the high cost of genetic testing, a lack of awareness about genetic services, and concerns about discrimination following genetic diagnosis have been identified as challenges to risk reduction in hereditary breast/ovarian cancer, particularly among low-income and minority populations with low rates of attendance for genetic counseling and testing, such as in the United States [22]. Affordability of genetic testing has also emerged as a concern in our study. Notably, the cost of genetic testing exceeded the monthly family income of 50% of the women who participated in telephone interviews. In Sri Lanka, genetic testing is not routinely offered in the state sector as part of free healthcare. Most patients pay out of pocket for private sector services [17]. Therefore, there may be value in developing a public health approach to identify high-risk individuals based on family and clinical history, particularly for those who cannot afford genetic testing. Offering targeted cancer surveillance to untested high-risk individuals in such a context could be a feasible strategy to enhance the early detection and management of hereditary breast cancer.

The majority of patients with a strong family history in our cohort were referred for genetic testing after developing at least one personal breast or ovarian cancer. This raises the question of whether these individuals would have benefited had they been referred for early genetic testing or cancer surveillance based on family history, before developing personal cancer. Although this study did not specifically investigate whether the breast cancers in these patients were detected early through opportunistic breast screening or diagnosed after the onset of signs and symptoms, it underscores the importance of emphasizing family history assessment at the primary care level for the early identification of pre-symptomatic high-risk individuals. The importance of family history as the starting point of genetic risk assessment has reached international consensus [1].

Failure to suspect an underlying genetic predisposition on the part of the clinicians may also result due to lack of clear guidelines for early identification & prompt referral of high-risk groups for genetic evaluation. The current Sri Lankan National Guideline on Cancer Early Detection and Referral Pathways of Common cancers for Primary Care Physicians, as outlined by the National Cancer Control Programme (NCCP), gives criteria for referral to genetic consultation, including criteria for identifying high-risk pre-symptomatic individuals based on family history [15]. However, the extent to which healthcare providers are aware of and adhere to these guidelines, and obstacles to referral remains uncertain. This once again underscores the need for a resource adapted local public health approach to enhance early identification of individuals at high risk. Rasing public awareness, and providing dedicated space in the available public health websites for identification and surveillance of high-risk individuals may be a feasible starting point towards early detection; we did not come across such dedicated local web pages for high-risk groups in the internet.

Despite the need for more intense breast cancer screening for *BRCA*-positive women been recognized in Sri Lanka [15], we observed that 42.8% of women in our cohort had not undergone any form of annual surveillance including CBE, mammography, or MRI. Additionally, none had received dual screening with both mammography and MRI.

We were unable to find similar regional or prior local studies discussing surveillance care received by women at high risk. Like Sri Lanka, India underscores prioritizing cancer screening to risk groups, however, no national guideline exits [23]. Breast imaging guidelines published by the Breast Imaging Society of India (2022) recommend annual screening mammography (and MRI) to *BRCA* positive women and their untested first-degree relatives starting at 30 years of age (or 10 years before the age of diagnosis of the first degree relative) [24], nevertheless, we could not find information on national or regional surveillance programs for high-risk women in India. This highlights the potential inadequacy of care received by high-risk women in the region.

This study did not delve into the reasons behind these women not undergoing surveillance. However, we note that although post-test counselling is received once by all *BRCA* positive individuals at HGU before reverting them to referring physicians, attendance for screening with CBE or mammography may not be ensured as there is no system for patient registration for sending reminders at least for hereditary breast cancer or high-risk groups. It is also observed that Sri Lanka cancer incidence and mortality data based on National Cancer Registry does not describe family history details for breast cancer [25]. Developing patient registration systems, data systems for high-risk groups and/or hereditary breast cancer may be of value in this regard. The current state sector opportunistic screening facilities do not provide screening MRI in Sri Lanka.

It is worth noting that the majority of study participants perceived themselves to be at a higher risk of developing breast cancer compared to the normal population. Furthermore, they expressed a willingness to participate in regular screening programs. It is also noteworthy that despite limited mammographic facilities in the country compared to high-income countries in the region, all patients in this study resided within 25 km of a state sector mammographic facility. These factors may suggest the potential for successful participation in a cancer surveillance program if offered. It is also important to highlight that although the majority were aware of being placed at an increased risk compared to the general population, most women were unaware of the actual magnitude of their lifetime risk of developing breast cancer, this could have been due to recall bias. The relationship between perceived risk and attendance at mammography screening programs has been reported with varying outcomes based on differing levels of risk perception [26]. Considering these findings, the development of patient registration systems coupled with screening invitations and reminders for high-risk women to attend available opportunistic screening services, may prove beneficial.

The primary limitation of this study is the small sample size, which raises a query about the underlying reasons for low case numbers of the genetic diagnosis in Sri Lanka, as discussed. Additionally, due to the small sample size, it was not feasible to estimate associations between practices and behaviors with socio-demographic factors as originally planned. Furthermore, reliance on telephone interviews to inquire about health behaviors is a notable limitation which introduces the possibility of recall bias and likely respondent exhaustion due to prolonged interviews, potentially impacting the reliability and validity of the results. To address these limitations, several measures were implemented. These included pre-interview training and piloting sessions, the collection of existing data from HGU records before conducting interviews to establish context and rapport with respondents, and scheduling interviews at times preferred by participants. It is important to note that this study did not assess cancer prevention strategies within the study group. Nevertheless, it is worth mentioning that none of the patients had undergone prophylactic mastectomy of the unaffected breast(s).

## Conclusion

In this study, we found that fifty percent of *BRCA1/2* gene-positive women had not received annual imaging-based surveillance by mammography or MRI, and none had undergone dual screening with mammography and MRI. This preliminary investigation, conducted in the context of Sri Lanka, raises a critical concern regarding the adequacy of breast cancer surveillance for genetically predisposed women in resource-constrained South Asian countries.

These findings underscore the need to develop locally adapted public health strategies that identify high-risk, individuals and provide them with regular coordinated surveillance. Given the limitations of resource-constrained settings, priority should be given to leveraging available opportunistic breast cancer screening services to ensure equitable care for this high-risk group.

## Data Availability

The datasets generated and/or analysed during the current study are not publicly available due the sensitive nature related to hereditary cancer genes in Sri Lanka but are available from the corresponding author on reasonable request.

## Abbreviations

BRCA: Breast cancer gene
CBE: Clinical breast examination
LI: Low income
LMI: Lower-middle income
HI: High income
WHO: World health organization
HGU: Human genetics unit
MRI: Magnetic resonance imaging
NICE: National institute for health and care excellence, UK
GCE: General certificate of education, Sri Lanka
BSE: Breast self-examination
NCCP: National cancer control programme of Sri Lanka
